# Correction: Radical ligand transfer: mechanism and reactivity governed by three-component thermodynamics

**DOI:** 10.1039/d4sc90254h

**Published:** 2025-01-07

**Authors:** Zuzanna Wojdyla, Martin Srnec

**Affiliations:** a J. Heyrovský Institute of Physical Chemistry, The Czech Academy of Sciences Dolejškova 3, Prague 8 18223 Czech Republic martin.srnec@jh-inst.cas.cz

## Abstract

Correction for ‘Radical ligand transfer: mechanism and reactivity governed by three-component thermodynamics’ by Zuzanna Wojdyla *et al.*, *Chem. Sci.*, 2024, **15**, 8459–8471, https://doi.org/10.1039/D4SC01507J.

In the published article, eqn (2) reads:
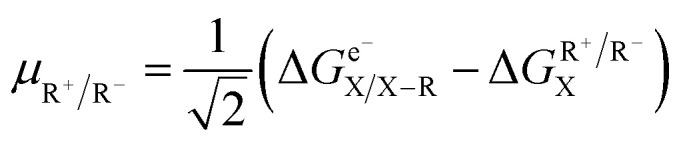
and contains a typographic error. The correct formula for *μ*_R^+^/R^−^_ is:
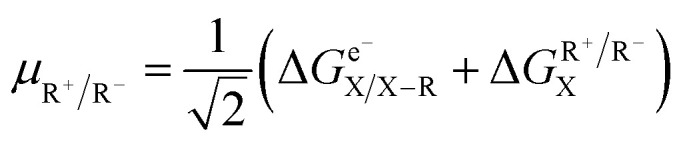
in line with ref. 64.

The values presented in the article were obtained using the correct equation.

The Royal Society of Chemistry apologises for these errors and any consequent inconvenience to authors and readers.

